# A preliminary prediction model for self-harm in adolescent inpatients with mental disorders: integrating individual and family factors

**DOI:** 10.3389/fpsyt.2026.1848287

**Published:** 2026-09-14

**Authors:** Shufen Wang, Yongna Wang, Jing Shao, Yan Wu, Qingyan Yang, Yanfang Zhou

**Affiliations:** Beijing Huilongguan Hospital, Capital Medical University, Beijing, China

**Keywords:** adolescents, nomogram, parental depression, psychiatric inpatients, self-harm

## Abstract

**Background:**

Self-harm is a major public health concern among adolescents, particularly those hospitalized with psychiatric disorders. Despite its clinical salience, risk stratification tools incorporating both individual-level and family-level factors remain limited. This study aimed to develop and preliminarily evaluate a prediction model for self-harm among adolescent inpatients with mental disorders.

**Methods:**

This cross-sectional study included 123 adolescent inpatients (aged 12–17 years). Self-harm was defined as intentional self-injury or poisoning regardless of suicidal intent. Demographic, clinical, and familial variables were extracted from electronic medical records. Psychological assessments included the Children’s Depression Inventory (CDI), Parent-child Relationship Scale, Parents’ Perception of Uncertainty Scale (PPUS), parental Generalized Anxiety Disorder-7 (GAD-7), and parental Patient Health Questionnaire-9 (PHQ-9). The dataset was split into a training set (80%, n = 98) and a test set (20%, n = 25). Logistic regression with backward stepwise selection was used to construct a nomogram, with model performance assessed by the area under the curve and decision curve analysis.

**Results:**

Four independent predictors of self-harm were identified: female sex (OR = 9.484, 95% CI: 2.736-32.868), higher CDI score (OR = 1.175 per 1-point increase, 95% CI: 1.058-1.305), higher parental PHQ-9 score (OR = 1.235 per 1-point increase, 95% CI: 1.064-1.433), and parental marital status (overall *P* = 0.001). The model showed good discrimination in the training set (AUC = 0.873) and acceptable performance in the testing set (AUC = 0.720). Decision curve analysis confirmed clinical utility across relevant threshold probabilities.

**Conclusion:**

Female sex, higher depressive symptom severity, higher parental depressive symptoms, and parental marital status were associated with self-harm among hospitalized adolescents with mental disorders. The developed nomogram may serve as a preliminary screening aid for individualized risk stratification and may help clinicians identify patients requiring further assessment. External validation in larger, multicenter cohorts is needed before routine clinical use.

## Introduction

Self-harm, defined as intentional self-poisoning or self-injury regardless of suicidal intent, represents a major public health concern among adolescents worldwide (1). Its prevalence is higher among adolescents with psychiatric disorders and is associated with suicide attempts and increased risk of premature mortality (2, 3). For adolescents with psychiatric disorders, identifying factors associated with self-harm may support early assessment, safety planning, and targeted intervention.

Adolescent self-harm is generally understood as the result of interactions among individual, clinical, and family-related factors (1, 4). Several theoretical perspectives support this view. Nock’s functional model suggests that self-harm may be maintained by intrapersonal and interpersonal reinforcement processes, including the regulation of negative affect and responses to interpersonal stress (5, 6). Ideation-to-action theories, such as the integrated motivational–volitional model and the three-step theory, further suggest that psychological distress, hopelessness, social disconnection, and capability for self-harm may influence the development of suicidal and self-harming behaviors (7, 8). In addition, a recent bio-ecological review of adolescent mental health identifies the family as a proximal context associated with adolescent emotional and behavioral outcomes (9).

Previous studies have identified depressive symptoms as one of the most consistent clinical correlates of adolescent self-harm and suicidal behavior (10, 11). Family-related factors have also been associated with self-harm risk, including parental psychopathology, family functioning, parent–child relationships, parental separation, and adverse family experiences (12–20).However, much of the existing evidence has been derived from community or school-based samples, and fewer studies have focused on adolescents with psychiatric disorders in inpatient settings. In addition, many studies have examined individual or family factors separately, making it difficult to determine their relative contributions when considered together.

Another gap concerns prediction modelling. Although prediction models may help clinicians estimate individualized risk, model development in clinical samples can be affected by limited sample size, sparse outcome events, and overfitting. Therefore, candidate predictors should be selected with theoretical and clinical justification, and model complexity should be considered in relation to the available number of self-harm events (21–23).The present study aimed to develop and preliminarily evaluate a prediction model for self-harm among hospitalized adolescents with psychiatric disorders. The specific objectives were: (i) to examine demographic, clinical, and family-related factors associated with self-harm; (ii) to identify independent predictors using logistic regression; (iii) to construct a nomogram for individualized risk estimation; and (iv) to evaluate model performance using internal validation.

## Materials and methods

### Study design and participants

This was a cross-sectional study of adolescent inpatients with mental disorders admitted to Beijing Huilongguan Hospital, Capital Medical University, between January 2024 and December 2024. Patients aged 12–17 years with complete clinical records were considered eligible. Individuals with severe cognitive impairment or inability to complete the self-report questionnaires were excluded. A total of 124 patients initially met the inclusion criteria; one patient was excluded due to missing data on the primary outcome (self-harm), resulting in a final analytic sample of 123 patients. The study was approved by the Institutional Review Board of Beijing Huilongguan Hospital, and informed consent was obtained from all participants and their legal guardians.

### Variables and measurements

Outcome variable: Self-harm was defined as intentional self-injury or overdose of medication, irrespective of suicidal intent, consistent with established epidemiological definitions. This broad definition was adopted to capture the overall spectrum of self-harm behaviors rather than restricting the outcome to a specific subtype. Accordingly, the outcome encompassed both suicidal and non-suicidal self-harm. In this study, self-harm was identified based on documented clinical records indicating any episode of self-injurious behavior (e.g., cutting, hitting) or intentional overdose of medication occurring within the three months prior to admission. Patients with no such history were classified as the non-self-harm group.

### Demographic and clinical characteristics

Demographic and clinical characteristics extracted from electronic medical records included age, sex, age at onset, illness duration, history of school bullying, sibling status, birth order, perceived family financial status, and admission diagnosis.

### Psychological assessments

Depressive symptoms were assessed using the Children’s Depression Inventory (CDI), a 27-item self-report scale with total scores ranging from 0 to 54; higher scores indicate more severe depression (Cronbach’s α = 0.87 in this study). The parent–child relationship was measured using the Chinese version of the Parent–child Relationship Scale (Parent Version) (24, 25). This 20-item scale is rated on a 5-point Likert scale (1–5) and encompasses four dimensions: emotional communication, independence and autonomy, friendship and intercourse, and mutual trust (Cronbach’s α = 0.87 in this study). Parental uncertainty regarding their child’s illness was assessed using the Chinese version of the Parents’ Perception of Uncertainty Scale (PPUS) (26), originally developed by Mishel (27). The scale comprises 28 items rated on a 5-point Likert scale, with four subscales (ambiguity, lack of information, lack of clarity, and unpredictability). Total scores range from 28 to 140, with higher scores indicating greater perceived uncertainty (Cronbach’s α = 0.85 in this study). Parental anxiety and depressive symptoms were measured using the Generalized Anxiety Disorder-7 (GAD-7) and the Patient Health Questionnaire-9 (PHQ-9), respectively. The GAD-7 is a 7-item scale with scores from 0 to 21 (Cronbach’s α = 0.89 in this study) (28), and the PHQ-9 is a 9-item scale with scores from 0 to 27(Cronbach’s α = 0.91 in this study) (29); higher scores on both instruments indicate more severe symptoms.

### Statistical analysis

All statistical analyses were performed using R software (Version 4.3.1). Before model development, the extent of missingness was examined for all candidate predictors extracted from electronic medical records. Variables with more than 30% missing values were excluded from the candidate predictor set. No missing data were present for the variables included in the final multivariable model; therefore, no imputation was performed and no participants were excluded from the final model because of missing data. Categorical variables were converted to factor variables. For categories with sparse observations (fewer than five) or those that were clinically or semantically similar (e.g., widowed and divorced under parental marital status) were collapsed into a single “Other” category to ensure model stability. Continuous variables were retained as numeric predictors, and those with zero variance were removed to eliminate non-informative features. Psychological scale scores were analyzed as raw values without standardization. The primary outcome, self-harm, was dichotomized as a binary factor (“No” indicating absence, “Yes” indicating presence).

To ensure cohort comparability, the dataset was split into a training set (80%) and an internal validation test set (20%) using stratified random sampling based on the outcome distribution. This yielded 98 observations (60 events) in the training set and 25 observations (15 events) in the test set. The training set was used for variable selection and model development, while the test set was reserved exclusively for internal validation.

Univariable logistic regression analyses were performed on the training set to identify potential predictors. Variables with a two-sided *P* < 0.10 were selected as candidates for multivariable modeling. Multicollinearity among CDI, PPUS, GAD-7, and PHQ-9 scores was assessed in the training set using tolerance and variance inflation factor (VIF) values. Given the limited sample size and number of events, the degrees of freedom in multivariable modeling were constrained by the number of events to reduce overfitting risk. Adhering to the widely recommended events-per-variable (EPV) criterion of at least 10 (30), the maximum number of candidate predictors was restricted to five. Backward stepwise likelihood ratio selection was applied for multivariable logistic regression, with a two-sided *P* < 0.05 as the criterion for final variable retention, to derive the optimal parsimonious predictive model. The linearity of continuous predictors with the logit of the outcome was assessed using the Box–Tidwell procedure. A nomogram was subsequently constructed based on the final multivariable model to facilitate individualized clinical risk assessment. The discriminative ability of the final model was evaluated using receiver operating characteristic (ROC) curves, and the area under the curve (AUC) with its 95% confidence interval (95% CI) was calculated for both the training and test sets. Decision curve analysis (DCA) was performed to evaluate the clinical utility of the nomogram by quantifying net benefit across a range of threshold probabilities, comparing the nomogram against two extreme reference strategies: assuming that all patients self-harm and that none do. Calibration was assessed using calibration plots, calibration-in-the-large (CITL), calibration slopes, and Brier scores. All statistical tests were two-sided, and a *P* < 0.05 was considered statistically significant unless otherwise specified.

## Results

### Baseline characteristics and dataset splitting

A total of 123 eligible adolescent inpatients with mental disorders were divided into a training set (n = 98) and a test set (n = 25) using stratified random sampling at an 8:2 ratio, with 60 and 15 self-harm events, respectively (**Table 1**).

**Table 1:** Comparison of general characteristics between the training set and the test set.

Variable	Training set (n=98)	Test set (n=25)	Statistics (t/z/χ2)	*P* value
Self-harm (Yes/No)^a^	60/38	15/10	0.013	0.911
Age (year, Mean ± SD)	15.23 ± 2.50	14.68 ± 1.65	1.053	0.295
Sex (M/F)^a^	29/69	9/16	0.383	0.536
Age of onset (year, Mean ± SD)	12.86 ± 1.97	12.88 ± 1.74	-0.053	0.958
Illness duration (month, Mean ± SD) ^b^	27.91 ± 19.96	26.52 ± 20.91	-0.342	0.732
School bullying (Yes/No)^a^	46/52	11/14	0.069	0.793
CDI score (Mean ± SD)	33.13 ± 6.16	32.20 ± 5.92	0.681	0.497
Siblings^a^			6.391	0.033
Only child	48	19		
two siblings	46	5		
Other (multiple siblings)	4	1		
Patient birth order^a^			1.676	0.430
First-born	81	21		
Second-born	16	3		
Other (third-born or later)	1	1		
Admission diagnosis^a^			3.071	0.350
Unipolar depression	66	16		
Bipolar disorder	14	2		
OCD	14	7		
Other (schizophrenia, etc.)	4	0		
Parental marital status^a^			1.820	0.493
First marriage	85	20		
Remarriage	5	3		
Other (widowed, divorced)	8	2		
Perceived family financial status^a^			1.614	0.847
Difficult Tense	5	1		
Tense	10	1		
Average	71	20		
relatively affluent	10	2		
Other (very affluent)	2	1		
Parent-child Relationship Scale score (Mean ± SD)	69.70 ± 11.64	75.68 ± 7.08	-3.248	0.002
Emotional communication	19.97 ± 4. 16	22.00 ± 3.07	-2.283	0.024
Mutual trust	15.02 ± 2.57	16.24 ± 2.15	-2.183	0.031
Friendship and intercourse	24.69 ± 4.27	26.68 ± 2.64	-2.913	0.005
Independence and autonomy	9.97 ± 2.09	10.52 ± 1.67	-1.220	0.025
PPUS total score	84.28 ± 8.99	81.96 ± 4.71	1.770	0.081
Ambiguity	32.71 ± 6.40	30.56 ± 4.94	1.567	0.120
Lack of information	14.43 ± 2.00)	13.80 ± 2.29	1.377	0.171
Lack of clarity	26.21 ± 3.13	26.28 ± 2.79	-0.096	0.924
Unpredictability	11.23 ± 2.43	11.44 ± 2.34	-0.383	0.702
Parental GAD-7 score^b^	5.62 ± 4.28	4.72 ± 3.18	-0.771	0.441
Parental PHQ-9 score^b^	5.54 ± 4.48	4.32 ± 3.76	-1.149	0.251

^a^ Categorical data for comparisons using 𝒳^2^ test.

^b^ Non-normally distributed continuous data for comparisons using Mann-Whitney U test. Continuous normally distributed data, analyzed by independent samples t-test. CDI: Children’s Depression Inventory. PPUS: Parents’ Perception of Uncertainty Scale. GAD-7: Generalized Anxiety Disorder-7. PHQ-9: Patient Health Questionnaire-9 items.

Significant differences were observed between the two sets in the total score and all four dimensions of the Parent-child Relationship Scale (all *P* < 0.05). No significant differences were found in other demographic, clinical, and scale-based baseline characteristics, including age, sex, age at onset, illness duration, school bullying, CDI score, PPUS total score, parental GAD-7, and PHQ-9 scores (all *P* > 0.05). No substantial multicollinearity was identified among CDI, PPUS, GAD-7, and PHQ-9 scores; tolerance values ranged from 0.420 to 0.916 and VIF values ranged from 1.091 to 2.383.

### Univariate logistic regression analysis (training set, n=98)

Univariable logistic regression identified multiple potential predictors of self-harm (**Table 2**). Female sex was a robust predictor (OR = 6.296, 95% CI:2.428-16.325, *P* < 0.001). Parental marital status showed an overall significant association (*P* = 0.011), though pairwise comparisons did not reach statistical significance (remarried vs. first marriage: OR = 0.467, 95% CI:0.074-2.940, P = 0.417; other vs. first marriage: OR not estimable due to sparse data).

**Table 2:** Univariable logistic regression model for self-harm (training set, n = 98).

Variable	Comparison	β	SE	OR (95% CI)	P
Age	Per 1-year increase	-0.008	0.083	0.992 (0.842 - 1.169)	0.928
Sex	Female vs. Male	1.840	0.486	6.296 (2.428 -16.325)	1.535×10^-4^
Age of onset	Per 1-year increase	0.040	0.105	1.041 (0.846 - 1.280)	0.706
Illness duration	Per 1-momth increase	-0.010	0.010	0.990 (0.971 - 1.011)	0.354
School bullying	No vs. Yes	-0.673	0.424	0.510 (0.222 - 1.173)	0.113
CDI score	Per 1-point increase	0.145	0.041	1.156 (1.067 - 1.253)	3.937×10^-4^
Siblings					0.353
	two siblings vs. Only child	0.559	0.428	1.749 (0.756 - 4.043)	0.191
	Other vs. Only child	0.932	1.190	2.538 (0.246 - 26.178)	0.434
Patient birth order					0.384
	Second-born vs. First-born	0.033	0.565	1.033 (0.342 - 3.126)	0.954
	Other vs. First-born	-16.044	1455.398	NE	0.991
Admission diagnosis					0.061
	Bipolar disorder vs. Unipolar depression	0.233	0.647	1.250 (0.352 - 4.440)	0.730
	OCD vs. Unipolar depression	-1.281	0.616	0.278 (0.083 - 0.929)	0.038
	Other vs. Unipolar depression	-1.792	1.184	0.167 (0.016 - 1.697)	0.130
Parental marital status					0.011
	Remarriage vs. First marriage	-0.762	0.939	0.467 (0.074 - 2.940)	0.417
	Other vs. First marriage	17.209	1398.721	NE	0.990
Perceived family financial status					0.136
	Tense vs. Difficult	1.792	1.208	6.000 (0.563 - 63.987)	0.138
	Average vs. Difficult	0.954	0.946	2.596 (0.407 - 16.564)	0.313
	Relatively affluent vs. Difficult	0.405	1.111	1.500 (0.170 -13.226)	0.715
	Other vs. Difficult	-15.161	1029.122	NE	0.988
Parent-child relationship scale score	Per 1-point increase	-0.009	0.018	0.991(0.957-1.027)	0.626
Emotional communication	Per 1-point increase	0.005	0.050	1.005(0.911 - 1.108)	0.927
Mutual trust	Per 1-point increase	-0.104	0.085	0.901(0.762 - 1.065)	0.222
Friendship and intercourse	Per 1-point increase	-0.018	0.049	0.982 (0.891 - 1.081)	0.710
Independence and autonomy	Per 1-point increase	-0.083	0.102	0.921(0.754 - 1.124)	0.417
PPUS total score	Per 1-point increase	0.066	0.026	1.069 (1.016 - 1.124)	0.011
Ambiguity	Per 1-point increase	0.080	0.036	1.083 (1.010 - 1.162)	0.026
Lack of information	Per 1-point increase	0.126	0.107	1.134 (0.919 - 1.399)	0.242
Lack of clarity	Per 1-point increase	0.149	0.073	1.161 (1.007 - 1.339)	0.040
Unpredictability	Per 1-point increase	0.029	0.086	1.029 (0.870 - 1.218)	0.737
Parental GAD-7 score	Per 1-point increase	0.099	0.056	1.105 (0.990 - 1.232)	0.074
Parental PHQ-9 score	Per 1-point increase	0.137	0.056	1.147 (1.027 - 1.281)	0.015

NE, not estimable due to sparse data (infinite confidence interval). SE, standard error. OR, odds ratio. CI, confidence interval. CDI: Children’s Depression Inventory. PPUS: Parents’ Perception of Uncertainty Scale. GAD-7: Generalized Anxiety Disorder-7. PHQ-9: Patient Health Questionnaire-9 items.

Continuous scale scores with significant associations included: CDI score (OR = 1.156 per 1-point increase, 95% CI: 1.067-1.253, *P* < 0.001); PPUS total score (OR = 1.069, 95% CI: 1.016-1.124, *P* = 0.011), with its subscale Ambiguity (OR = 1.083, 95% CI: 1.010-1.162, *P* = 0.026) and Lack of clarity (OR = 1.161, 95% CI: 1.007-1.339, *P* = 0.040); and parental PHQ-9 score (OR = 1.147, 95% CI: 1.027-1.281, P = 0.015). Parental GAD-7 score showed a borderline significant association (OR = 1.105, 95% CI: 0.990-1.232, *P* = 0.074).

Admission diagnosis had a borderline overall association (*P* = 0.061); specifically, obsessive‑compulsive disorder was associated with lower odds of self‑harm compared with unipolar depression (OR = 0.278, 95% CI:0.083-0.929, *P* = 0.038). No significant associations were found for age, age at onset, illness duration, school bullying, sibling status, birth order, perceived family financial status, or any subscale of the Parent-child Relationship Scale (all *P* > 0.05).

Variables with *P* < 0.10 (sex, CDI score, parental marital status, PPUS total score, parental PHQ-9 score were selected as candidates for multivariable modeling. Although admission diagnosis (overall *P* = 0.061) and parental GAD‑7 score (*P* = 0.074) also met the screening threshold, they were not included owing to the events‑per‑variable constraint (maximum five predictors).

### Multivariable logistic regression analysis (training set, n=98)

Based on the univariable analysis results, sex, CDI score, parental marital status, PPUS total score, and parental PHQ-9 score were initially entered into the multivariable logistic regression model. After backward stepwise selection, PPUS total score was excluded due to lack of statistical significance (*P* = 0.175), suggesting that its univariable association was largely explained by other covariates. The final model retained four variables: sex, CDI score, parental PHQ-9 score, and parental marital status (**Table 3**). The Box–Tidwell test showed no significant violation of the linearity-in-the-logit assumption for CDI score (*P* = 0.575) or parental PHQ-9 (*P* = 0.602).

**Table 3:** Final multivariable logistic regression model for self-harm (training set, n = 98).

Variable	Comparison	β	SE	OR (95% CI)	P
Sex	Female vs. Male	2.250	0.634	9.484 (2.736 - 32.868)	3.890×10^-4^
CDI score	Per 1-point increase	0.161	0.054	1.175 (1.058 - 1.305)	0.003
Parental marital status	–	–	–	–	0.001
	Remarried vs. First marriage	-1.459	1.087	0.233 (0.028 - 1.956)	0.179
	Other vs. First marriage	18.339	1829.874	NE	0.992
PHQ-9 score	Per 1-point increase	0.211	0.076	1.235 (1.064 - 1.433)	0.005

NE, not estimable due to sparse data (infinite confidence interval); SE, standard error; OR, odds ratio; CI, confidence interval; CDI, Children’s Depression Inventory; PHQ-9, Patient Health Questionnaire-9 items. *P* value for parental marital status is the overall *P* value for the variable.

In the final model, female sex was significantly associated with self‑harm (OR = 9.484, 95% CI: 2.736-32.868, *P* < 0.001). Each 1-point increase in CDI score was associated with an 18% increase in the odds of self-harm (OR = 1.175, 95% CI: 1.058-1.305, *P* = 0.003). Each 1‑point increase in parental PHQ‑9 score was associated with a 23% increase in the odds (OR = 1.235, 95% CI: 1.064-1.433, *P* = 0.005). Parental marital status was significantly associated with the outcome overall (overall *P* = 0.001). Compared with parents in their first marriage, those who were remarried had lower odds of child self-harm, although the pairwise comparison did not reach statistical significance (OR = 0.233, 95% CI: 0.027-1.957, *P* = 0.179), and the “other” category (widowed and divorced combined) yielded an unstable estimate due to sparse data.

### Nomogram for predicting self-harm

A nomogram integrating the four independent predictors from the final multivariable model was constructed (**Figure 1**), enabling quantitative individualized risk assessment of self-harm. For clinical illustration, a female patient with a CDI score of 36 and a parental PHQ-9 score of 8 (assuming parents in their first marriage) had an approximate 70% predicted risk of self-harm. This nomogram provides a user-friendly visual tool for clinicians to identify high-risk patients and initiate targeted intervention.

**Figure 1:**
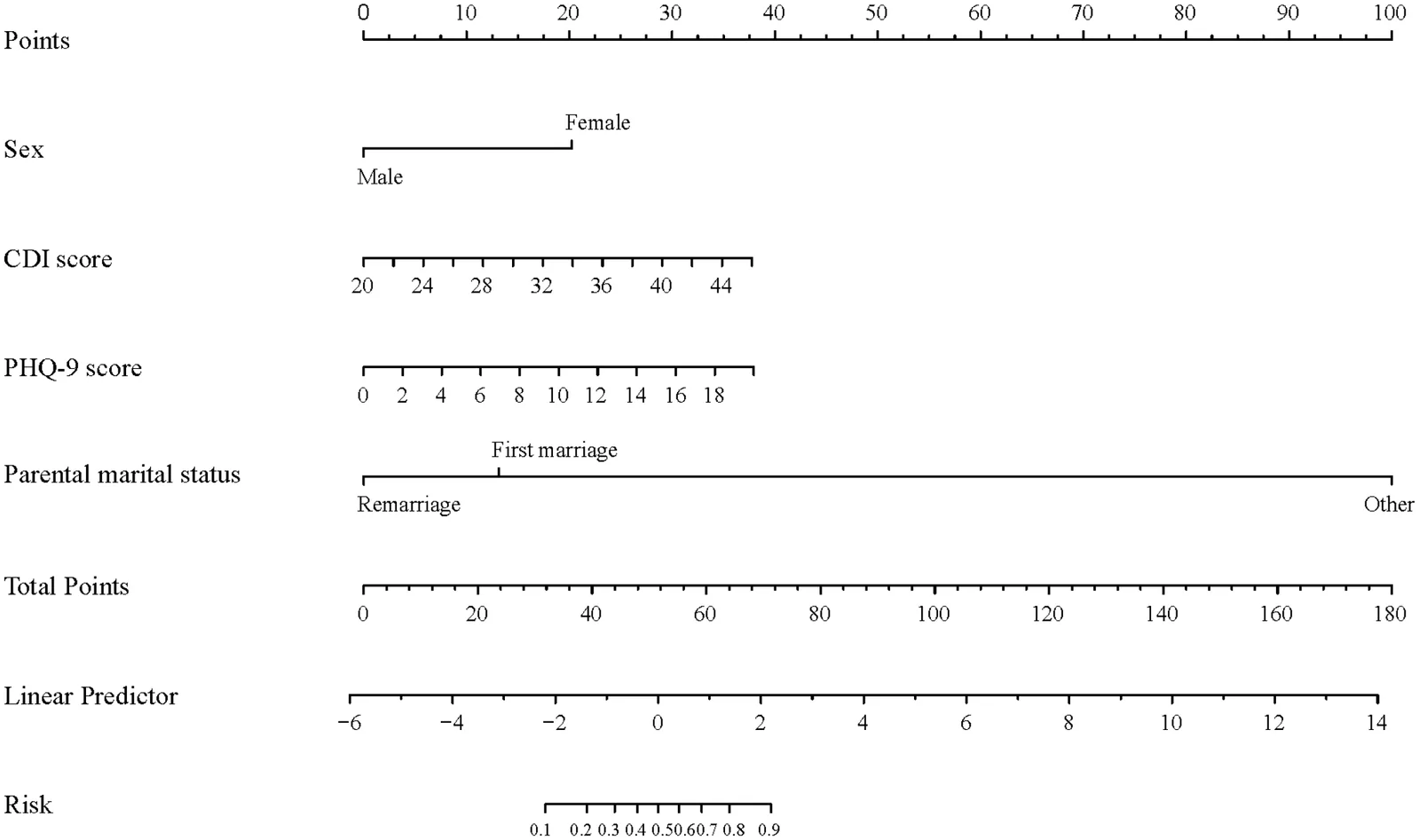
Nomogram for predicting the risk of self-harm in hospitalized adolescent psychiatric patients. Nomogram incorporating sex, Children’s Depression Inventory (CDI) score, parental Patient Health Questionnaire-9 items (PHQ-9) score, and parental marital status. To obtain the predicted probability of self-harm, locate each predictor on its respective axis, draw a vertical line upward to the “Points” axis to obtain the corresponding points for each variable, sum the total points, locate the total on the “Total Points” axis, and draw a vertical line downward to the “Risk” axis for the final predicted risk.

### Model performance and internal validation

ROC curve analysis was used to evaluate the discriminative ability of the final model in both the training and test sets (**Figures 2A, B**). The model exhibited good discriminative ability in the training set (AUC = 0.873, 95% CI:0.803-0.943). In the independent test set, the model demonstrated moderate discriminative ability (AUC = 0.720, 95% CI:0.491-0.949). The lower AUC in the test set is likely attributable to the small sample size (n = 25), a common limitation in internal validation of predictive models with limited events. Nevertheless, the model still showed acceptable predictive performance in this independent cohort. DCA confirmed the clinical utility of the nomogram (**Figure 2C**). In the training set (solid blue line), the nomogram provided a positive net benefit across threshold probabilities of approximately 0.10-0.65. In the testing set (solid red line), the nomogram demonstrated superior net benefit compared with the “treat all” strategy across threshold probabilities of approximately 0.36-0.50. The Brier scores were 0.140 in the training set and 0.236 in the test set. In the training set, the CITL was approximately 0 and the calibration slope was 1.000, whereas in the test set, the CITL was 0.549 and the calibration slope was 0.030. Calibration plots for the training and test sets are presented in **Figure 3**.

**Figure 2:**
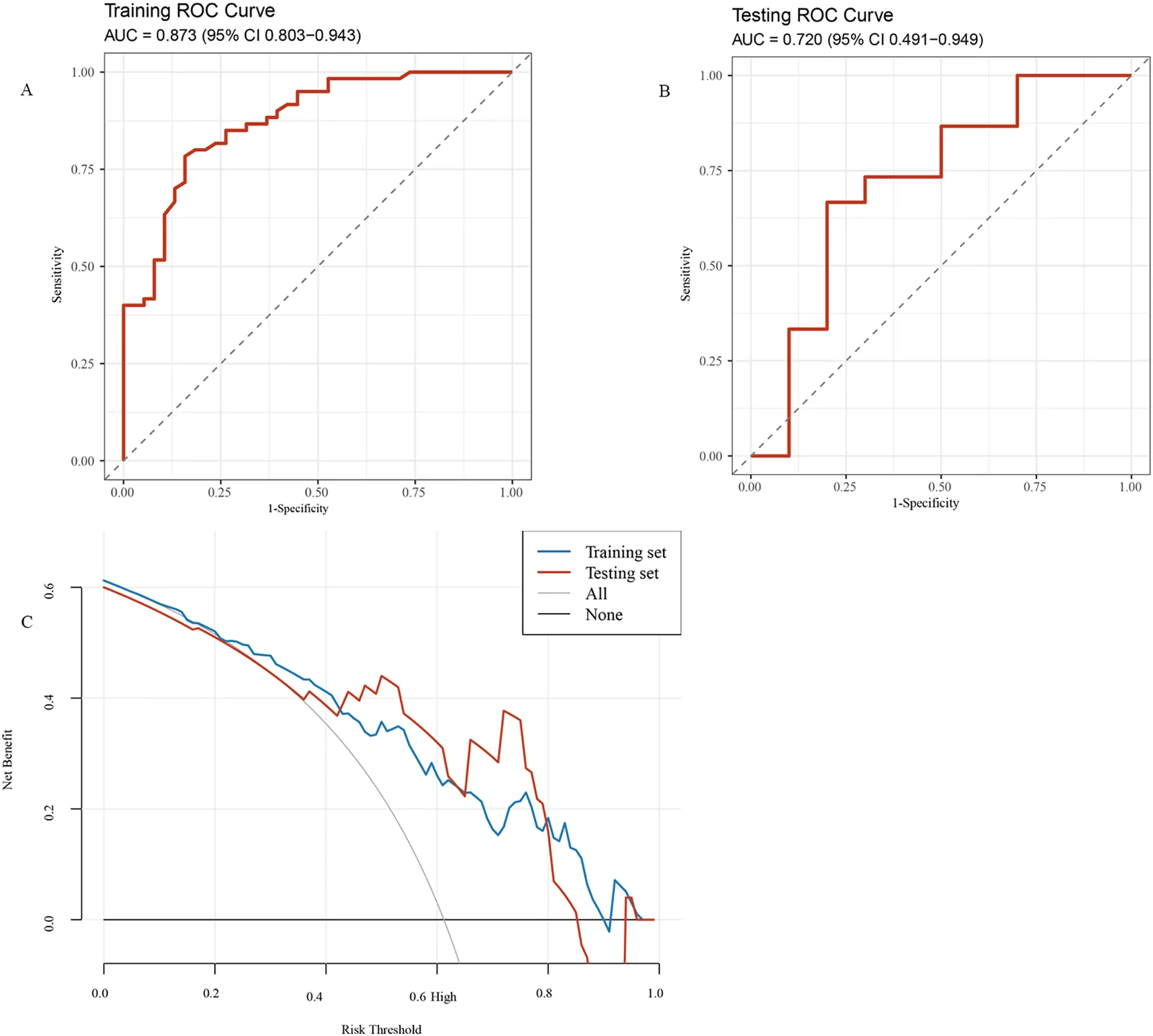
Receiver operating characteristic (ROC) curves and decision curve analysis (DCA) of the final multivariable logistic regression model. **(A)** Training set (n=98): AUC = 0.873, 95% CI:0.803-0.943; **(B)** Test set (n=25): AUC = 0.720, 95% CI:0.491-0.949. **(C)** Decision curve analysis. Solid blue line: training set (n = 98); solid red line: testing set (n = 25); gray solid line: “treat all” strategy; black horizontal line: “treat none” strategy (net benefit = 0). The nomogram provides a positive net benefit across threshold probabilities of approximately 0.10-0.65 (training set) and demonstrates superior net benefit compared with the “treat all” strategy across approximately 0.36-0.50 (testing set). AUC: area under the curve; CI: confidence interval.

**Figure 3:**
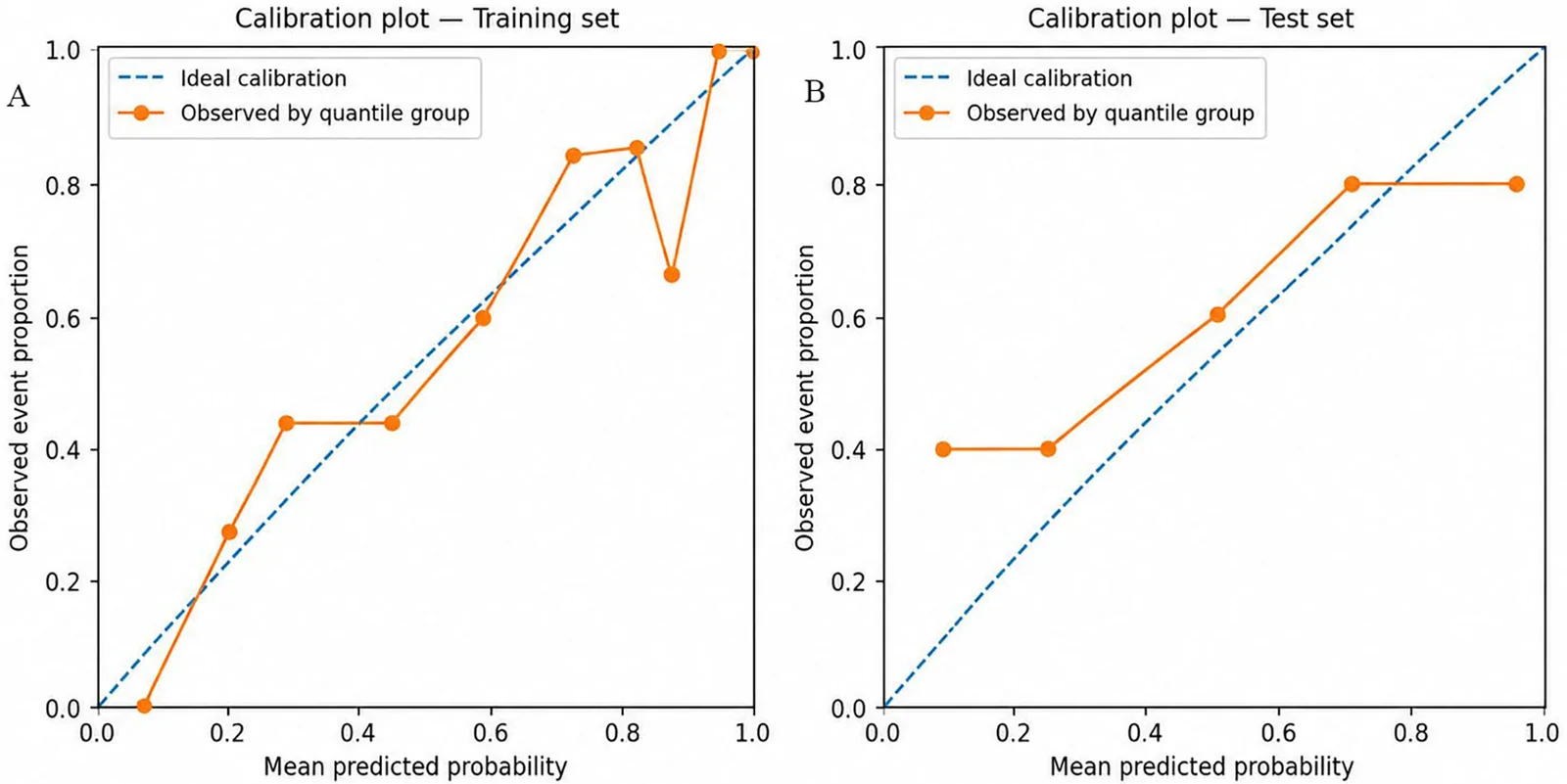
Calibration plots of the prediction model in the training and test sets. **(A)** Training set; **(B)** Test set. The dashed diagonal line represents perfect calibration.

## Discussion

In this study of 123 hospitalized adolescents with psychiatric disorders, we developed a preliminary multivariable prediction model for self-harm integrating individual- and family-level factors and evaluated its performance in a held-out test set. Using rigorous univariable and multivariable logistic regression with strict events-per-variable constraints, we identified four independent predictors emerged: female sex, higher depressive symptom severity (CDI score), higher parental depressive symptoms (parental PHQ-9 score), and parental marital status. These factors formed a clinically interpretable nomogram, which showed good discrimination in the training set (AUC = 0.873) and a test-set AUC of 0.720. However, given the small test sample and uncertainty in model performance, these findings should be considered preliminary and require confirmation in larger external cohorts.

Our finding that female sex was strongly associated with self-harm is consistent with the well-documented gender disparity in adolescent self-harm (1) particularly in clinical populations where females present with higher rates of internalizing disorders and emotion dysregulation (31, 32). Meta-analytic evidence confirms that female adolescents are at significantly higher risk of self-harm than their male peers, with the gender disparity being particularly pronounced in clinical samples where psychiatric comorbidity is more prevalent; although the magnitude of this difference varies across studies, the meta-analysis reported an overall odds ratio of 1.50, which increased to 2.25 in clinical populations (31). In the present study, the observed association between female sex and self-harm (OR ≈ 9.5) was notably stronger than estimates typically reported in community-based samples, which may reflect the higher baseline risk and greater clinical complexity characteristic of inpatient populations.

Depressive symptomatology is consistently identified as a key correlate of self-harm among adolescents (1, 33). In our study, each one-point increase in the CDI score corresponded to an 18% increase in the odds of self-harm, underscoring the dose‑response nature of this relationship. This finding aligns with prior evidence showing that depressive symptom severity predicts suicide attempts in adults (34) and that symptom improvement predicts reduced self-harm behaviors in adolescents with major depressive disorder (35).

Admission diagnosis showed a borderline overall association with self-harm in univariable analysis (P = 0.061). Patients with obsessive‑compulsive disorder (OCD) had significantly lower odds of self-harm than those with unipolar depression (OR = 0.278, 95% CI: 0.083-0.929, P = 0.038), consistent with prior evidence that the association between OCD and suicidality is largely accounted for by comorbid depression severity (36). Larger studies are needed to further explore diagnostic subgroup differences.

Contrary to expectations, neither the total score nor any subscale of the Parent-child Relationship Scale was significantly associated with self-harm. Several factors may account for this null finding. First, in a hospitalized sample with high clinical complexity, parent‑child relationship quality may be uniformly compromised, limiting its discriminative ability. Second, the scale was completed by parents; discrepancies between parent and adolescent perceptions of family functioning are well documented (37), and adolescent perceptions may show stronger associations with self‑harm outcomes. Third, the impact of family environment may be mediated by parental mental health. Indeed, parental depressive symptoms and marital status emerged as significant predictors, suggesting that parental emotional well‑being and family structure are more proximal determinants of risk than relationship quality per se in this acute clinical context.

In this study, parental depressive symptoms, as measured by the PHQ-9, emerged as an independent predictor of adolescent self-harm, even after adjusting for patients’ own depression severity and family structure (parental marital status). Each one-point increase in parental PHQ-9 score was associated with a 23% increase in the odds of self-harm. This finding is consistent with prior evidence highlighting the critical role of familial factors, including parental mental health, in shaping adolescent self-harm risk (12), which may involve genetic vulnerability and impaired parent‑child interaction. Extending prior work showing that family relationship factors remain associated with adolescent suicidality after controlling for parental and adolescent psychopathology (38), our study demonstrates that parental depressive symptoms contribute independently to self-harm risk in adolescent inpatients with mental disorders. This underscores the importance of assessing parental mental health as part of routine clinical evaluation in inpatient psychiatric settings.

Parental marital status was retained in the final model and showed an association with adolescent self-harm. However, this finding should be interpreted cautiously, as pairwise comparisons were not statistically significant and some subgroup estimates were unstable due to sparse data. Clinically, parental marital status may serve as a proxy indicator of broader family-related psychosocial context, including family stability, caregiving continuity, parent–child communication, emotional support, and exposure to family conflict or life stressors, rather than representing a direct causal factor. This interpretation is consistent with previous evidence showing that poor family functioning is positively associated with adolescent non-suicidal self-injury (39). The association remained after adjustment for parental depressive symptoms, suggesting that parental marital status may capture family-related risk beyond current parental mental health. Future studies with larger samples and more detailed assessments of family functioning are needed to clarify the underlying mechanisms. Although the PPUS total score was significantly associated with self-harm in univariable analysis, it was excluded during backward stepwise selection, likely reflecting collinearity with parental depressive symptoms (PHQ‑9). Parents with greater illness‑related uncertainty may experience higher levels of distress, which is consistent with prior research demonstrating associations between parenting stress, parental psychological distress, and adverse child outcomes in pediatric chronic illness populations (40).

The decline in AUC from the training set (0.873) to the test set (0.720) warrants consideration. While the small test set (n = 25) contributes to this instability, such attenuation is also characteristic of optimism in model development within single‑center cohorts (41). This underscores the need for external validation in larger, independent cohorts before clinical implementation. Nevertheless, decision curve analysis confirmed the nomogram’s clinical utility, demonstrating a positive net benefit across clinically relevant threshold probabilities. Calibration estimates in the test set deviated substantially from their ideal values; however, these estimates should be interpreted cautiously given the small test sample of 25 patients. These findings further support the preliminary nature of the model and the need for larger studies with more robust validation before clinical application.

Several limitations should be acknowledged. The sample size was limited, with 123 participants and 75 self-harm events, and the 80/20 split resulted in a small test set and some baseline imbalance between the two subsets, which may reduce the stability and precision of performance estimates. A single random split is also less efficient in a dataset of this size, and resampling-based approaches such as bootstrap validation or repeated cross-validation may provide more robust internal validation in future studies. Although model complexity was constrained according to the available number of events, the events-per-variable principle alone does not establish an adequate sample size for prediction model development. Sparse data in some categorical predictor levels also resulted in unstable or non-estimable odds ratios, further limiting the precision of the regression estimates. Moreover, univariable screening followed by backward stepwise regression may increase the risk of selection instability and overfitting; future studies with larger samples should consider penalized regression and shrinkage approaches. Therefore, the present model should be considered preliminary and its performance interpreted cautiously. The single-center design further limits generalizability. The cross-sectional design prevents causal inference, and the identified predictors should therefore be interpreted as associated factors rather than causal determinants. In addition, reliance on electronic medical records may introduce incomplete documentation and reporting bias. Although variables with more than 30% missing values were excluded and no missing data were present for the final model variables, retrospective data collection may still affect measurement consistency. Because self-harm was defined irrespective of suicidal intent, suicidal and non-suicidal behaviors were combined into a single outcome, which may obscure differences in their associated risk factors and limit the specificity of interpretation. Future studies should evaluate these outcomes separately. Parental marital status should also be interpreted cautiously as a crude proxy for broader family-related psychosocial context. Future models may benefit from incorporating more detailed psychosocial and mechanistic variables, such as adolescent mentalization or reflective functioning, which can be assessed using validated instruments such as the Three-Factor Reflective Functioning Questionnaire for Youth (42). Mentalization-based interventions, including group-based approaches, may also be considered in future intervention-oriented studies (43). Finally, external validation was not performed, and the nomogram should be considered a preliminary screening aid until validated in larger, multicenter, and more diverse populations. From a clinical perspective, the nomogram may serve as a preliminary screening aid for individualized risk stratification rather than a standalone decision-making tool. As shown in **Figure 1**, the model integrates routinely available variables, including sex, CDI score, parental PHQ-9 score, and parental marital status, which can be obtained during the initial clinical assessment or extracted from electronic medical records. In clinical workflows, the nomogram may help clinicians identify patients who warrant further comprehensive risk assessment, enhanced safety planning, closer observation, parental psychoeducation, or family-based interventions. In resource-constrained settings, it may provide a simple and structured approach to support risk-based triage when specialist assessment resources are limited. However, the predicted risk should be interpreted alongside clinical judgment, and any risk threshold should be determined according to local resources, clinical priorities, and further external validation.

## Conclusion

In this single-center study of 123 adolescent inpatients with mental disorders, female sex, higher depressive symptom severity, higher parental depressive symptoms, and parental marital status were associated with self-harm in the final multivariable model. The model showed good discrimination in the training set and a test-set AUC of 0.720; however, the performance estimates, including calibration, should be interpreted cautiously given the limited overall sample size and the small test set. Accordingly, the nomogram should be regarded as a preliminary prediction model rather than a clinically validated risk-assessment tool. Its performance and generalizability require confirmation through larger, multicenter studies with robust internal and external validation. Future prospective studies should also incorporate additional domains, such as childhood trauma, peer relationships, and detailed family functioning, to further clarify factors associated with adolescent self-harm.

## Data Availability

The datasets presented in this article are not readily available due to ethical restrictions approved by the Institutional Review Board of Beijing Huilongguan Hospital, Capital Medical University and institutional policies regarding sensitive patient information from a psychiatric inpatient setting. Requests to access the datasets should be directed to the corresponding author.
